# Ipsiversive ictal eye deviation in inferioposterior temporal lobe epilepsy—Two SEEG cases report

**DOI:** 10.1186/s12883-017-0811-8

**Published:** 2017-02-21

**Authors:** Wei Zhang, Xingzhou Liu, Lijun Zuo, Qiang Guo, Qi chen, Yongjun Wang

**Affiliations:** 1 Department of Neurology, Beijing Tiantan Hospital, Capital Medical University, Beijing, China; 2 Epilepsy Center, Guangdong Sanjiu Brain Hospital, Jinan University, No. 578, Sha Tai Nan Lu, Guangzhou, 510510 China; 3 School of Psychology, South China Normal University, Guangzhou, China; 4 China National Clinical Research Center for Neurological Diseases, Beijing, China; 5 Department of Neurology, Tiantan Clinical Trial and Research Center for Stroke, Beijing Tiantan Hospital, Capital Medical University, Beijing, China; 6 Vascular Neurology, Department of Neurology, Beijing Tiantan Hospital, Capital Medical University, Beijing, China

**Keywords:** Ipsiversive eye deviation, Inferioposterior temporal epilepsy, MT/MST complex, Anterior occipital sulcus

## Abstract

**Background:**

Versive seizure characterized by conjugate eye movement during epileptic seizure has been considered commonly as one of the most valuable semiological signs for epilepsy localization, especially for frontal lobe epilepsy. However, thelateralizing and localizing significance of ictaleye deviation has been questioned by clinical observation of a series of focal epilepsy studies, including frontal, central, temporal, parietal and occipital epilepsy.

**Case presentation:**

Two epileptic cases characterized by ipsiversive eye deviation as initial clinical sign during the habitual epileptic seizures are presented in this paper. The localization of the epileptogenic zone of both of the cases has been confirmed as inferioposterior temporal region by the findings of ictalstereoelectroencephalography (SEEG) and a good result after epileptic surgery. Detailed analysis of the exact position of the key contacts of the SEEG electrodes identified the overlap between the location of the epileptogenic zone and human MT/MST complex, which play a crucial role in the control of smooth pursuit eye movement.

**Conclusion:**

Ipsiversive eye deviation could be the initial clinical sign of inferioposterior temporal lobe epilepsy and attribute to the involvement of human MT/MST complex, especially human MST whichwas located on the anterior/dorsal bank of the anterior occipital sulcus (AOS).

## Background

Epileptic version or versive seizure, which has been defined as sustained and extreme conjugate eye movements with lateral head and body movements [1, 2], can occur during partial epileptic seizures. Contraversive epileptic eye deviation, often termed as “versive seizure”, is one of the most common types of frontal lobe seizure in which frontal eye field is involved by epileptic stimulation [3, 4]. Moreover, it is considered as one of the most valuable semiological signs for lateralization of epileptogenic zone [5–7].

However, the lateralizing and localizing significance of ictal eye deviation, even the versive type, during partial epileptic seizures has been questioned by clinical observations froma series of focal epilepsy studies focusing on the lateralization, particularly because of that the epileptic eye deviation may be ipsilateral or contralateral to the electroencephalography (EEG) focus, and has been associated with focal manifestations of EEG or neuroimaging evidence from frontal, central, temporal, parietal and occipital areas [8–22]. Although the fundamental reasons for these differences may be much more complicated than people originally understood, variations of eye movementsinduced by different pathophysiology during seizures must be considered.

So far, five categories of eye movements have been observed in human or non-human primates: pursuit, saccadic, vergence, vestibulo-ocular and optokinetictypes [23]. Among them, smooth pursuit is the most common type of eye movement guided by retinal imaging which mediates eye deviation to the visual stimuli [23, 24]. Although epileptic ipsiversion occurs incidentally and has been discussed curtly, ithasbeen attributed to activation of the smooth pursuit eye movements mediated by the temporo-occipital cortex [8, 9]. By contrast, epileptic contraversion was assumed to occur as a result of the stimulation of the saccadic system by the cortico-superior collicular pathway [8, 9].

In macaque monkeys, the middle temporal (MT) and medial superior temporal (MST) areas, which play an indispensablerole in normalsmooth pursuit movement, locate in the inferior posterior and medial superior temporal lobe, respectively [25–27]. It has been also observed that lesions of MT produce retinotopic deficits in the initiation of pursuit eye movement [28], and lesions of MST also produce directional deficits that are especially pronounced during maintained pursuit [29]. These results highlight a general distinction between the two areas: MT is largely involved in pursuit initiation, whereas MST is important for pursuit maintenance [24].

Only a few comparative studies hitherto investigated human homology of MT/MST functional organization, and the resultsindicated that the vicinity of posterior branch of the inferior temporal sulcus is motion-sensitive area and direct stimulation to this area induces constant ipsilateral eye deviation [25, 30, 31]. Anatomical correlation of the eyemovement disorders during epileptic seizuresgenerated in human MT (or MST), however, has been scarcely discussed [8, 13, 32]. We present here two caseswhose epileptogenic zones have been confirmed by ictal stereoelectroencephalography (SEEG) and freedom from seizures during the long term follow-up after surgery.

## Cases presentation

Case-1 was a 24-year old and right-handed man with no personal or family risk factors of epilepsyand febrile seizures. The initial epileptic seizuresoccurredat the age of 18, which were described as generalized tonic-clonic type (GTCS), at a frequency of three or four times a year. He was treated with oxcarbazepine and levetiracetam withno response. After his age of 22, he experienced “minor seizures” which were characterized by eyes and head deviation to his right followed by bilaterallyasymmetric and tonic limb posturing lasting about thirty seconds. The seizure frequency increased progressively from once a week to several tens a day without any triggering factor.

**Fig. 1:**
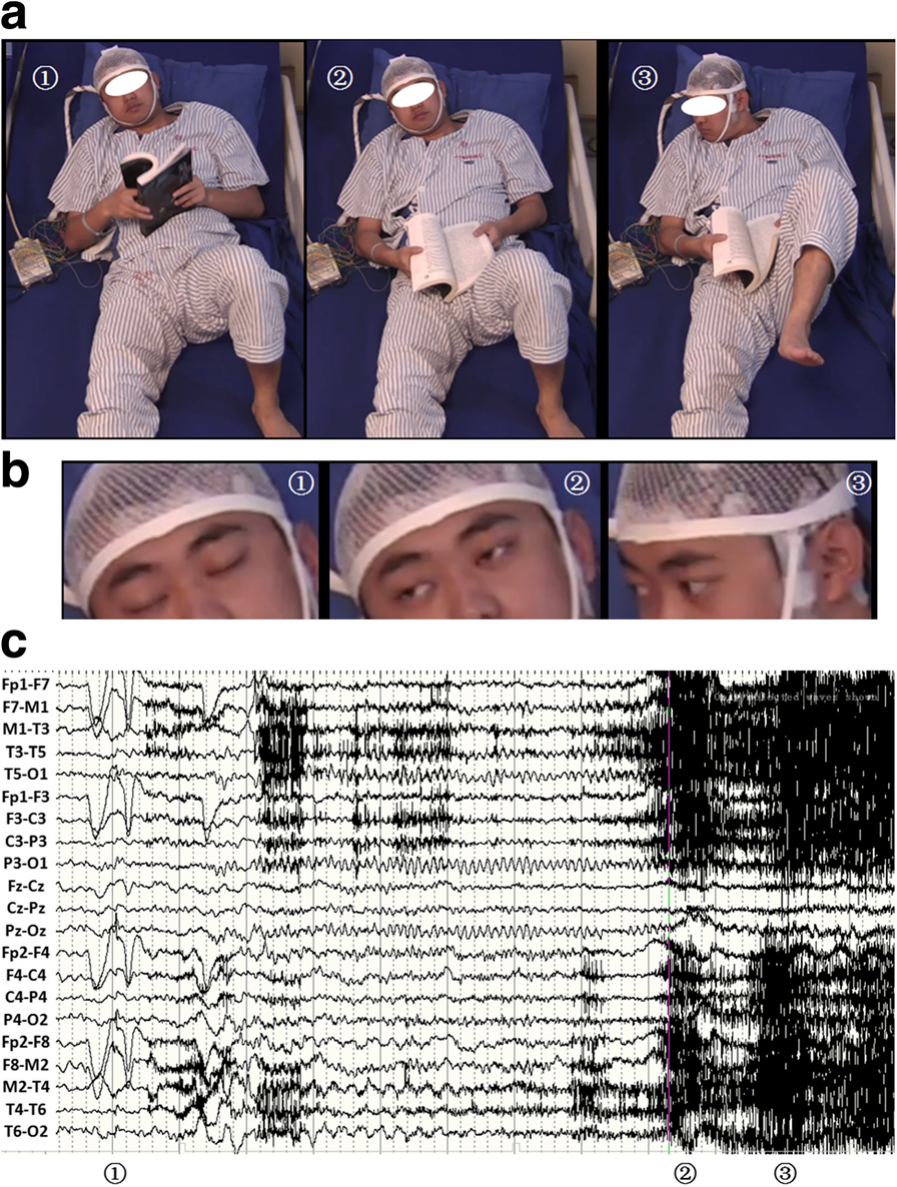
Electro-clinical semiology on non-invasive Video-EEG of Case-1. **a** The chronological semiology of habitual epileptic seizure without GTCS; **b** The close-up image of eyes and head of the same seizure; **c** Ictal non-invasive EEG of the same seizure. The sequential ictal clinical sign manifested on the imagine labeled by ① ~ ③: imagine ①shows eyes uncomfortable sensation and eyes close during 0-10 s of clinical seizure; imagine ② shows eyes forced right deviation on the time point of 9 s of clinical seizure; imagine ③ shows Eyes and head forced deviation to right side with left leg tonic posture during the 11 to 22 s of clinical seizure

**Fig. 2:**
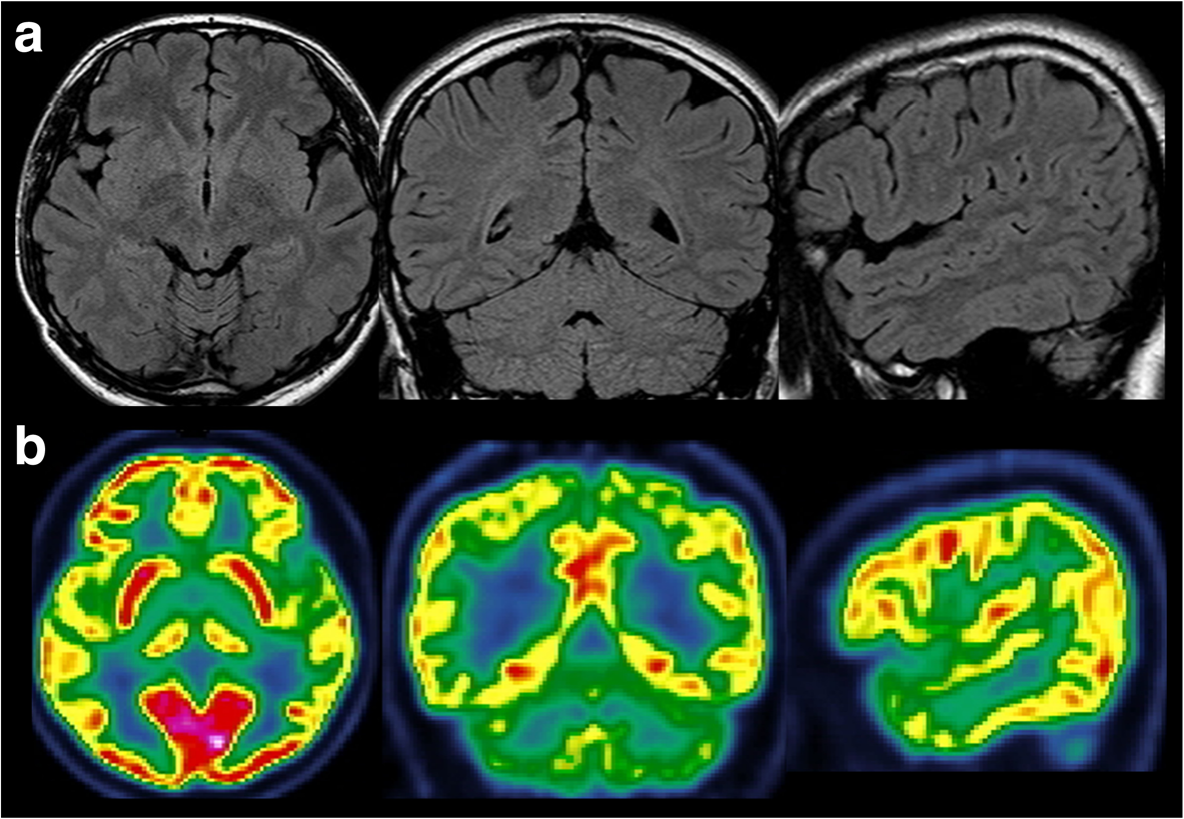
**a** and **b** showed the Brain MRI and FDG-PET of Case-1. No evaluable changes were identified for localization of epileptogenic lesion

Paroxysmal delta activityfollowed byspike and waves activity had been detected on right temporo-occipital region in the interictal scalp EEG. During the 3 days of scalp VEEG monitoring, a total of 32 habitual seizures were recorded, each of them lasted for 10 to 30 s and two of them were followed by secondary GTCS. The semiological chronology of the seizures can be summarized as forced eyedeviation to the right followed by right side turning ofthehead, left leg tonic posturing, left version and GTCS. The ictal EEG showed rhythmic theta-delta discharges followed by spike and waves activity over the right temporo-occipital region (Fig. 1). For presurgical evaluation, he underwent twoMRI scans, 1.5 and 3.0 tesla respectively, which were performed with 3 mm thickness with no interval, and a positron emission tomography with neurotracer of fluorodeoxyglucose 18 F (FDG-PET). Both of the MRI and FDG-PET scans were unremarkable (Fig. 2).

All the SEEG electrodeswith multiple contacts (10 to 15 contacts, length: 2 mm, diameter: 0.8 mm, 1.5 mm apart) were implanted with assistance of ROSA robot (Medtech, Montpellier, France). This procedure was preceded by a 3 tesla MRI scan performed with 1 mm thickness without interval for the implantation planning. A postoperative computed tomography (CT) scan without contrast was then used to verify both the absence of bleeding and the precise location of each contact. Finally, image reconstructionwas made in ROSA operating system to locate each contact anatomically along each electrode trajectory.

The patient underwent two different surgeries for SEEG implantation over his right hemisphere, and the second one, being performed 4 months after the first procedure, was in order to define the electrophysiological boundary of epileptogenic zone.

**Fig. 3:**
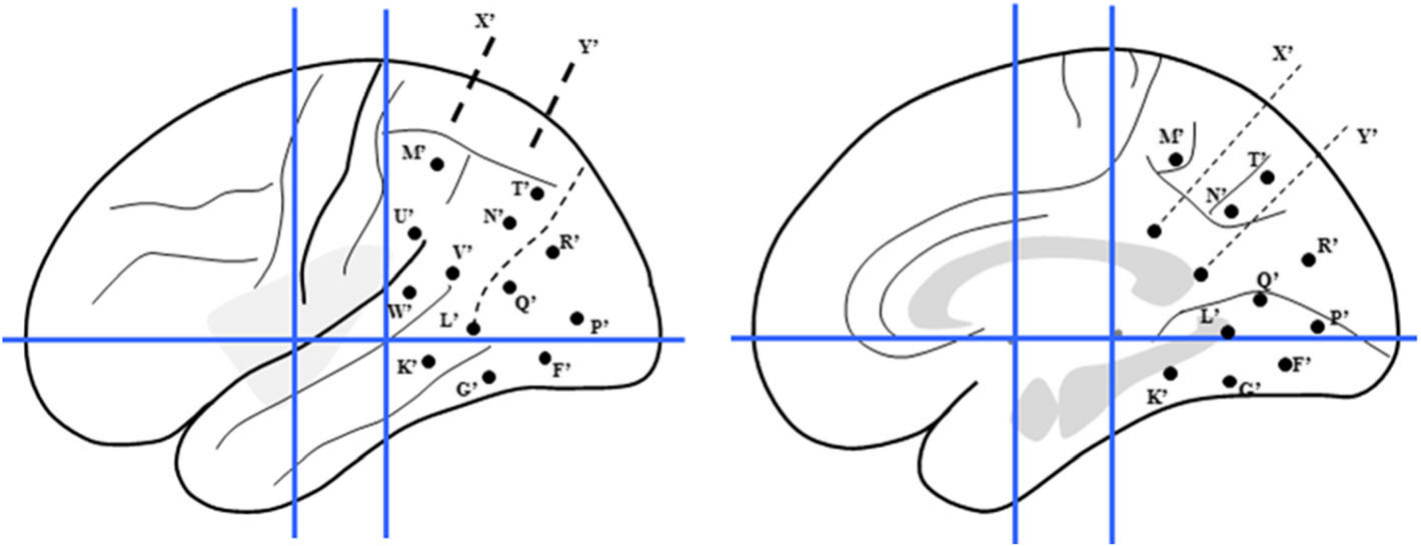
Sechematic diagram of the first SEEG electrode implantation of Case-1 on lateral and medial view of the Talairach’s basic referential system. X’: electrode exploring the dorsal part of posterior cingulatedgyrus (PCC) (medial contacts) and the anterior superior parietal lobule (SPL) (lateral contacts); Y’: electrode exploring the ventral part of PCC (medial contacts) and posterior SPL (lateral contacts); M’: electrode exploring anteriorprecuneus (medial contacts) and supramarginalgyrus (lateral contacts); T’: electrode exploring posterior precuneus (medial contacts) and angular gyrus (AG) (lateral contacts); N’: electrode exploring posterior precuneus on anterior bank of parieto-occipital fissure (medial contacts) and AG (lateral contacts); U’: electrode exploring SMG with lateral contacts; W’ and V’: electrodes exploring posterior part of superior temporal gyrus (STG) adjacent to superior temporal sulcus (STS) with lateral contacts; L’: electrode exploring the anterior part of lingual gyrus (LG) (medial contacts) and posterior segment of inferior temporal sulcus (ITS) (lateral contacts); K’: electrode exploring posterior parahippocampalgyrus (medial contacts) and middle temporal gyrus (MTG) (lateral contacts); G’: electrode exploring posterior fusiform gyrus (FG) (medial contacts) andposterior inferior temporal gyrus (ITG); Q’: electrode exploring LG (medial contacts) and anterior bank of ascending limb of ITS (lateral contacts); F’: electrode exploring LG (medial contacts) and posterior bank of ascending limb of ITS (lateral contacts); P’: electrode exploring LG (medial contacts) and convexity of occipital lobe (lateral contacts); R’: electrode exploring cuneusgyrus (CG) (medial contacts) and convexity of occipital lobe (lateral contacts)

**Fig. 4:**
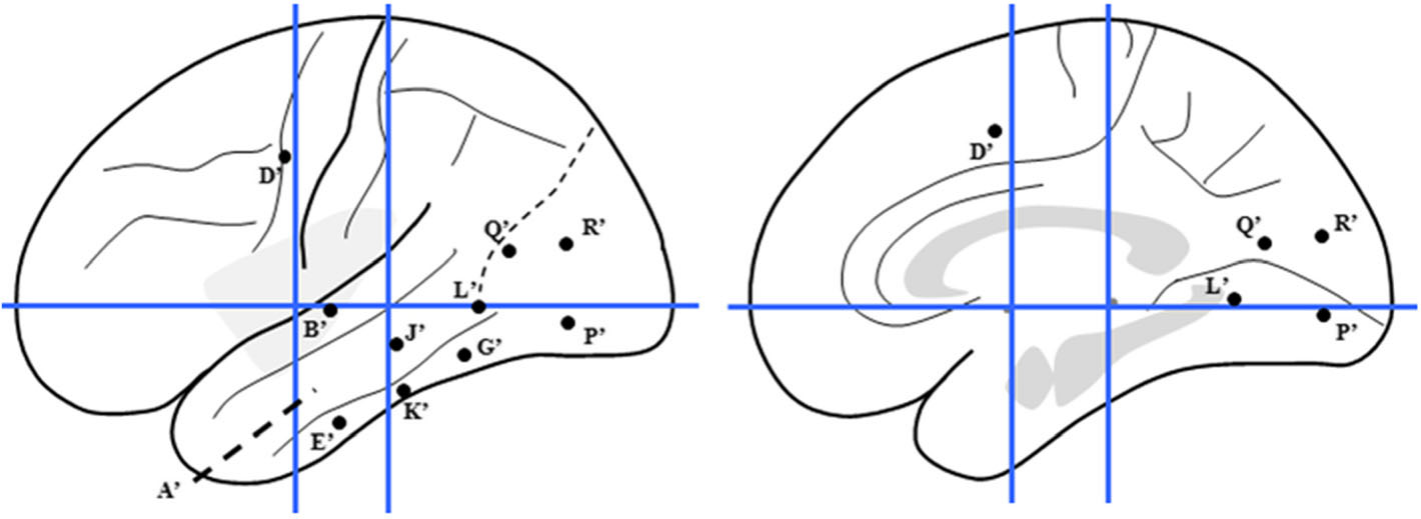
Sechematic diagram of the second SEEG electrode implantation of Case-1 on lateral and medial view of the Talairach’s basic referential system. G’: electrode exploring the posterior part of ITGwith lateral contacts and fusiform gyrus with medial contacts; L’: electrode exploring the posterior segment of ITS with lateral contacts and anterior LG with medial contacts; Q’: electrodeexploring the anterior bank of the anterior occipital sulcus (AOS) with lateral contacts and anterior bank of parieto-occipital fissure (POF) with medial contacts; P’ and R’: electrodes exploring the posterior bank of AOS with lateral contacts and lingual and CG with medial contacts; K’: electrode exploring the middle part of ITG with lateral contacts and collateral sulcus with medial contacts; E’: electrode exploring the anterior ITG with lateral contactsandentorhinal cortex with medial contacts; J’: electrodeexploring MTG with lateral contacts and hippocampus with medial contacts; A’: electrode exploring the temporal pole with lateral contacts and amygdale with medial contacts; B’: electrode exploring STG with lateral contacts and anterior insular long gyrus with medial contacts; D’: electrode exploring the frontal eye field (FEF) with lateral contacts and cingulate cortex with medial contacts

The first implantation covered extensive cortical areas related to eye movements as shown in Fig. 3, including intraparietal sulcus (IPS, anterior and posterior respectively), lateral (superior and inferior respectively) and medial parietal regions, lateral and medial occipital regions, occipito-parietaland occipito-temporal junctionas well as posterior part of medialand neocortical temporal regions. According to the findings of the first SEEG recording, the secondimplantationfocused on the cortical areas of early spreadingand surrounding areas as shown in Fig. 4 including limbic and neocortical temporal areas, temporo-occiptial areas, lateral and medialoccipitalareas.

**Fig. 5:**
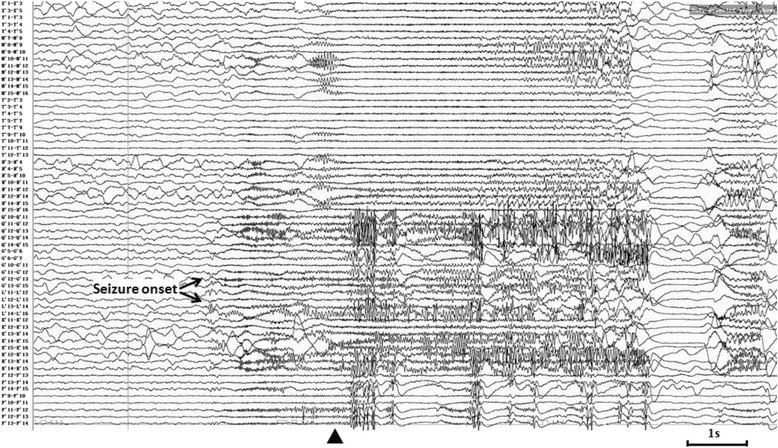
Ictal SEEG after the first electrodes implantation of Case-1. Seizure-onset was on G’12-13 and L’12-13 exploring the posterior part of inferior temporal area. The initial eyes left version appeared at the time point labeled by ▲

**Fig. 6:**
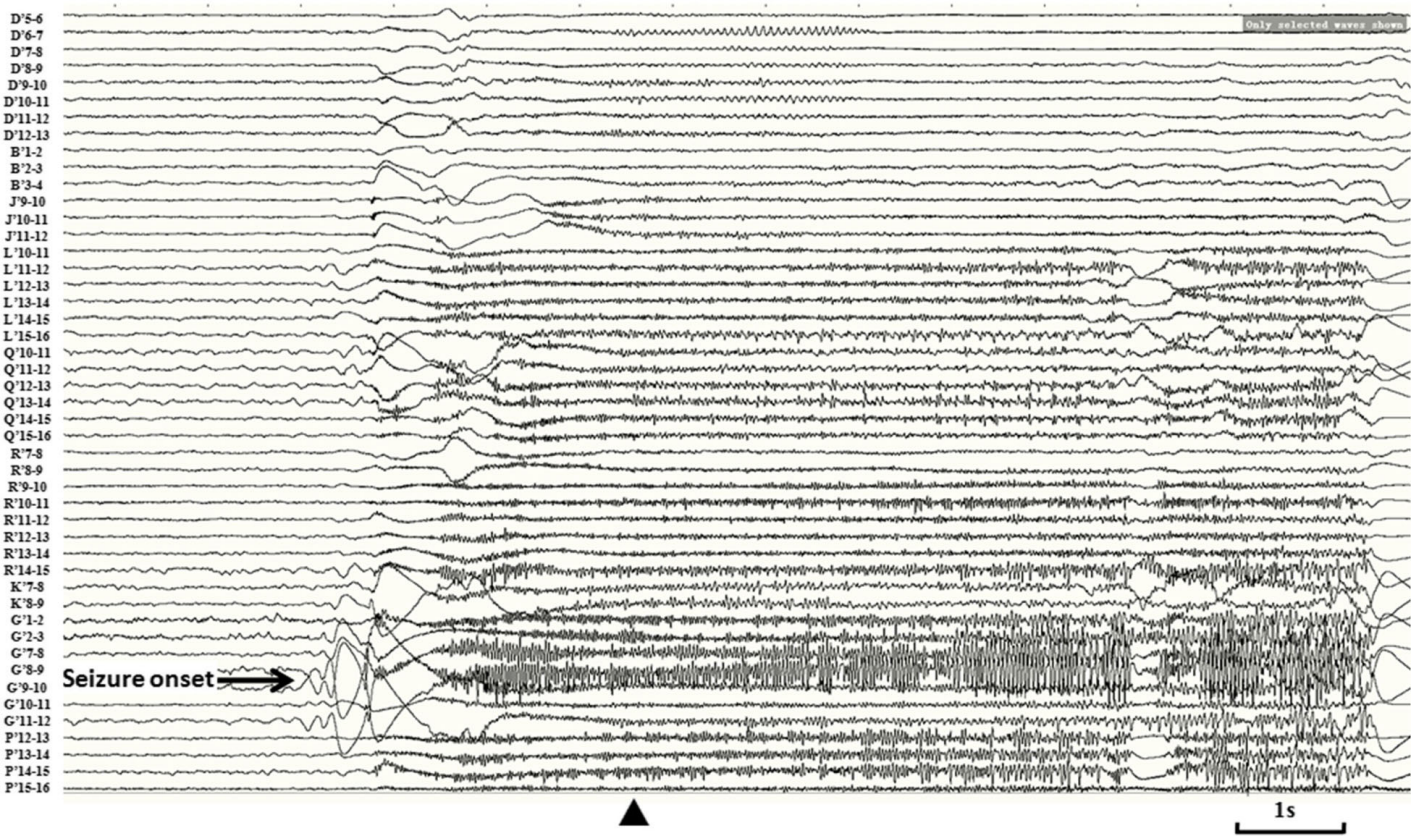
Ictal SEEG after the second electrodes implantation of Case-1. EEG seizure onset characteristic as initial slow followed by high frequency oscillations (HFO) was over lateral contacts of electrode G’ (G’9 and 12), which exploring the posterior ITG. The wide propagation of HFO involved multiple cortical areas, including posterior segment of ITS (L’13-16), anterior and posterior bank of AOS (Q’10-16, P’12-16), LOS (R’10-15), and even the location of FEF (D’5-12), within 400 ms

**Fig. 7:**
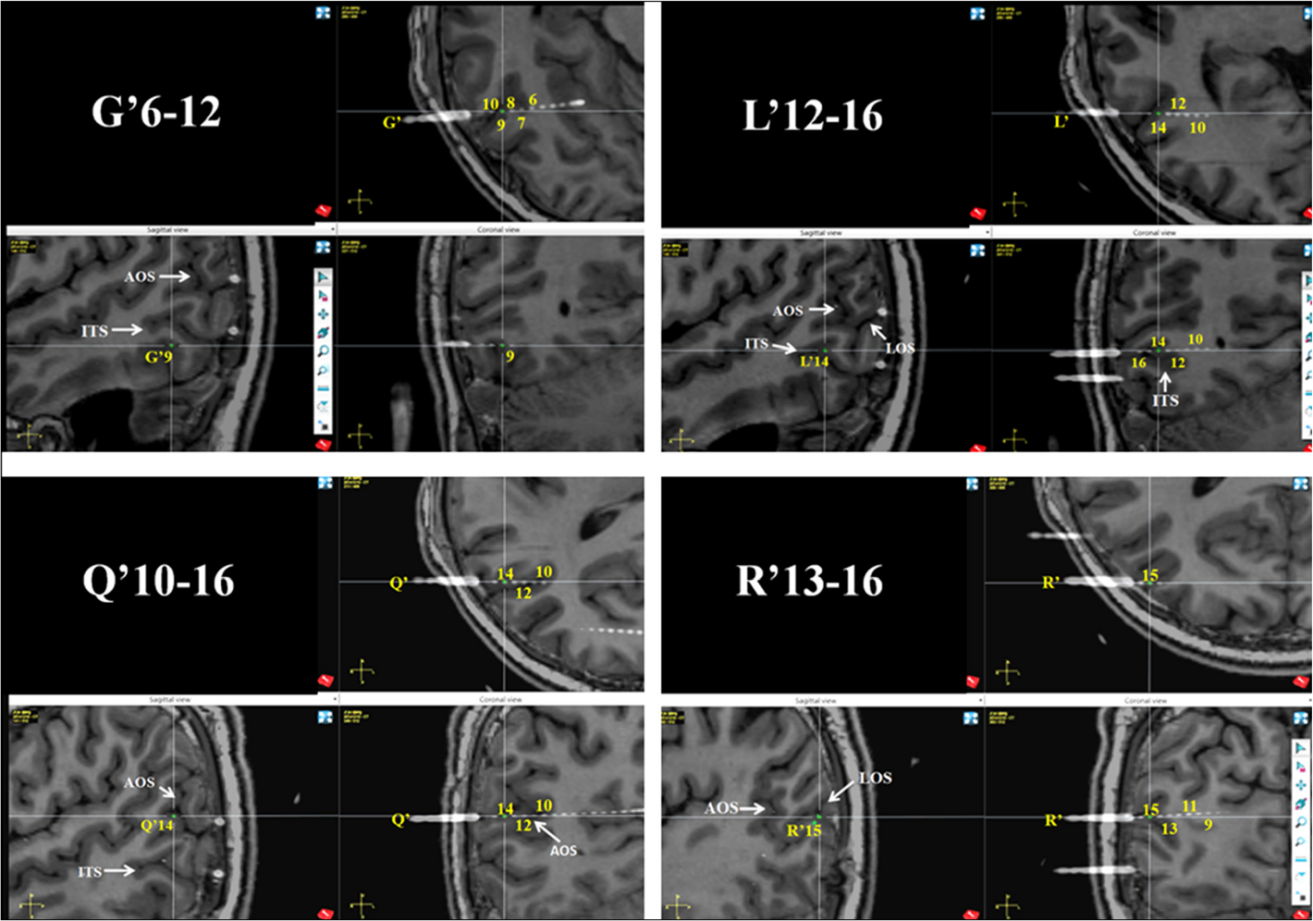
The reconstruction of the exact location of key electrodes through the fusion of postoperative CT and preoperative MRI data. AOS: anterior occipital sulcus; ITS: inferior temporal sulcus; LOS: lateral occipital sulcus

Resembling interictal pattern was obtained from both of SEEG recordings. Continuous or subcontinuous delta activities and 1.5-2Hz spike-waves were recorded from the inferoposterior temporal neocortex adjacent to the posterior segment of right inferior temporal sulcus (ITS) during interictal SEEG recording. Both of the SEEG recordingshowed similar initial epileptic discharges characterized as polyspikes followed by high frequency oscillations on the inferoposterior temporal regions. On the first ictal SEEG recording in which more extensive cortical areas were explored, the high frequency oscillations propagated to wide cortical areas including the temporo-occipital area, supramarginal and angular gyri, intraparietalsulcus within 600 ms (Fig. 5). The ictal SEEG of the second SEEG recording demonstrated the initial discharge originating from posterior inferior temporal gyrus (ITG) (contacts of G’8-9 and G’12), and the propagations of the high frequency oscillation involved the posterior segment of the ITS, anterior and posterior banks of anterior occipital sulcus (AOS), and lateral occipital sulcus (LOS), and even the location offrontal eyes field (FEF) within 400 ms (Fig. 6). The exactlocation of the key electrodesof the second SEEG electrodes implantation, obtainedfrom postoperative CT and preoperative MRI data fusion, has been shown on Fig. 7.

**Fig. 8:**
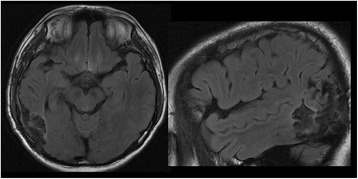
Postsurgical MRI of Case 1 and seizure freedom has lasted for 25 months since cortical resection

The location of the seizure onset zone was confirmed over the inferotemporal region through both of the SEEG recordings, and particularly during the second SEEG. The early spreading areasof electrical seizure were located over the posterior segment of ITS, anterior and posterior bank of AOS, and extended to cortical area adjacent to the posterior segment of superior temporal sulcus (STS). The cortical resection (Fig. 8) was done and includethe posterior part of the ITG and middle temporal gyrus (MTG), anterior and posterior banks of the AOS, and posterior segment of theSTS. Seizure-freedom lasted 17 months since epileptic surgery.

Case-2 was a 19-year old right-handed man with normal psychomotor development. When he was 3 years old, his parents observed several episodes of abnormal behaviors during sleep, which manifested as eyes open and staring for several seconds. The treatment of Phenobarbital led to a period of seizure-freedom for 1.5 years. Seizure recurred at the age of five after Phenobarbital withdrawn, and manifested as eyes staring and making fist of right hand lasting for 1 min with frequency of 1–2 times per day. Intentional response with external stimuli was lost during the episodes of seizures and recovered immediately after seizures. The patient reported his habitual epileptic aura as a kind of visual illusion “mimicking watching 3D movie”.

**Fig. 9:**
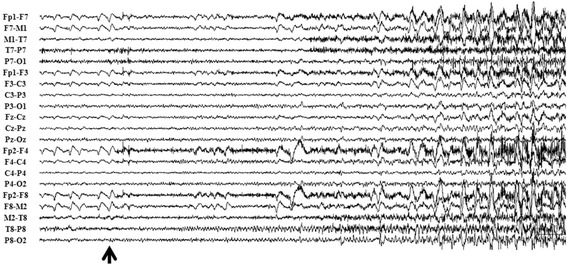
Ictal scalp-EEG of Case 2 showed rhythmic spikes on right middle-posterior temporal region at EEC onset (labeled by *black arrow*)

**Fig. 10:**
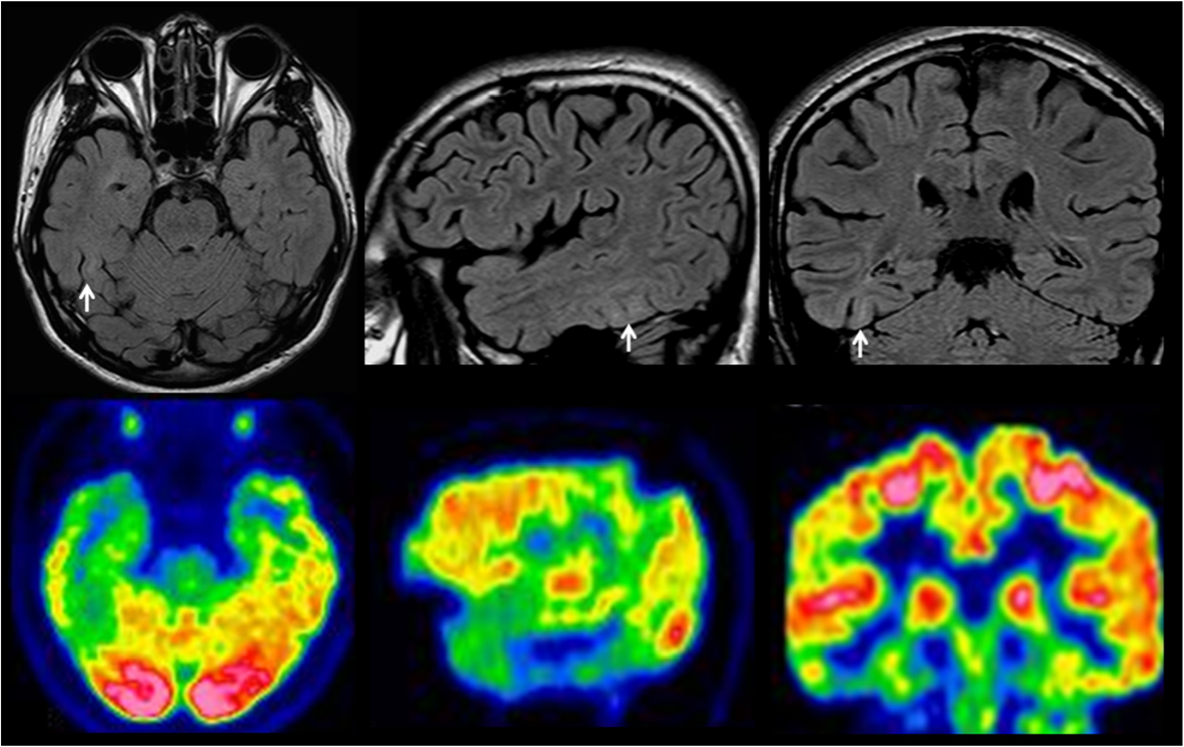
Brain MRI and PET scans of Case 2. T2flair imagins of brain MRI showed the high abnormal signals involving right middle-posterior part of ITG, lateral occipito-temporal sulcus and fusiform gyrus. PET scan demonstrated the hypometabolism regions over inferior and basal aspects of right temporal lobe, as well as the medial temporal structure

Interictal scalp-EEG showed numerous spike-waves on right temporal region with the highest amplitude of single spike over the middle temporal region. Video EEG captured three habitual seizures. The typical chronological semiology could be concluded was eyes open and staring followed by forced deviation to right side, left arm tonic posturing proximally with left fist clenched, and then left facial clonia only in one seizure. Each seizure lasted for about tens of seconds and no more than 1 min with loss of consciousness. EEG onset characterized by rhythmic spikes on right middle-posterior temporal region showed by ictal scalp-EEG (Fig. 9). Brain MRI scanning demonstrated high abnormal signals involving right middle-posterior part of ITG, lateral occipito-temporal sulcus and fusiform gyrus on T2 and T2flair slices (Fig. 10), which was presumed as the epileptogenic lesion by us. Hypometabolism on brain FDG-PET scanning pointed to the same region and supported our hypothesis (Fig. 10).

**Fig. 11:**
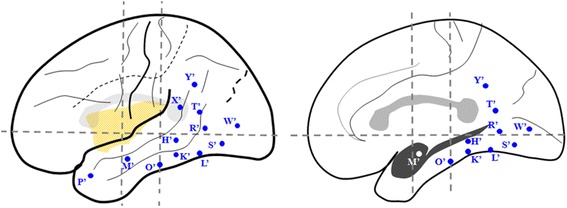
Sechematic diagram of the second SEEG electrode implantation of Case-2 on lateral and medial view of the Talairach’s basic referential system. K’ and L’: electrodes exploring the lesion on the middle and posterior part of ITG with lateral contacts and fusiform gyrus with medial contacts; O’ and S’: electrodes exploring the anterior and posterior border of the lesion identified by the MRI with lateral contacts and parahippocampalgyrus and fusiform gyrus respectively with medial contacts; H’ and R’: electrodes covered the middle and posterior MTG with lateral contacts and fusiform gyrus with medial contacts; T’: electrodecovered the angular gyrus with lateral contacts and ventral part of posterior cingualtegyrus with medial contacts in oblique orientation; Y’: electrode exploring the supramarginalgyrus with lateral contacts and dorsal part of posterior cingulategyrus with medial contacts; X’: electrode covered the angular gyrus with lateral contacts; W’: electrode exploring the most posterior part of ITG just anterior to the AOS with lateral contacts and lingual gyrus with medial contacts; M’: electrode exploring the anterior part of MTG with lateral contacts and amygdala with medial contacts; P: electrode covered the temporal pole withlateral and medial contacts

According to our consideration, the SEEG should have been performed in order to ascertain the causal relationship between the epileptic seizure and the putative epileptogenic lesion, and confirm the involvement of MT+ during the episodes of epileptic eye movements. The electrodes were placed to explore the following cortical foci: theright middle and posterior neocortical temporal cortex, temporal pole and medial structure (Fig. 11).

Constant polyspikes and ripple were identified over the fusiform gyrus (L’1-3, H’1-3, K’1-2) and posterior ITG (S’7-9) in interictal, as well as preictal phase of SEEG. The electro-clinical semiology obtained from SEEG was stereotyped and identical with that recorded by scalp-EEG. The putative lesion had been confirmed by ictal SEEG as epileptogenic lesion and the most posterior cortical part of ITG, which occupied the anterior bank of the AOS, was involved by ictal discharges within 5 s before the appearance of initial clinical sign—the ipsilateral eye deviation. The cortical resection had been guided by the neuroimaging and clinical neurophysiologic data, which had been described above in detailed. The core of the resection is the lesion identified by MRI, and the anterior border wasdetermined by the lateral contacts of electrode O’, posterior border was anterior bank of AOS, the superior borderreach the cortical areas explored by lateral contacts of the electrodes H’ and R’, and medial border is the collateral sulcus. The patient has been seizure-free for 25 months since the epileptic surgery without any remarkable neurological and neuropsychological deficit.

## Discussion

Epileptic eye deviation in seizure originated from the parieto-temporo-occipital region had been reported previously [8–16]. The underlying mechanisms of lateralized eye deviation during epileptic stimulation had been presumed as below: contralateral eye deviation is attributed to the involvement of cortical saccadic areas, and the stimulation of smooth pursuit cortical areas during epileptic seizures causesipsilateral ocular deviation [8, 9, 14, 33]. However, actual case of epileptic ipsiversion, manifesting as eyes conjugate deviation to the ipsilateral side of the epileptic focus, was rarely reported [33], and empirical evidence on the presumed mechanisms underlying the ipsilateral eye deviation has not been documented in details.

Eyes pursuits are smooth tracking movements which maintain foveal fixation when viewing a moving object and hence stabilize the retinal image, and the stimulus for pursuit is motion of an object. In macaque cerebral cortex, area MT complex (MT+), which includes the middle temporal (MT) and medial superior temporal (MST) areas, has been considered strongly direction-selective, and important in processing neuronal signals related to visual motion [34–36]. According to the neurophysiologic data from macaque, the neural pathway of pursuits originates in the primary visual cortex, and the projections are then sent to the extrastriate V5 which includes the areas of MT and MST [37–39]. The receptive field of area MT primarily includes the contralateral visual field, while area MSTd (dorsal MST) has receptive field that extends well into the ipsilateral visual field [26, 40]. Area MT neurons respond only when retinal motion is present [41, 42], and lesions of MT produce retinotopic deficits in the initiation of pursuit eye movement [28]. In contrast, MST neurons maintain their responses to object motion even when there is no retinal counterpart [41, 42], and lesions of MST produce directional deficits that are especially pronounced during maintained pursuit [29], also known as an ipsilateral pursuit deficit [29, 43].

The existence of area V5/MT+ has been demonstrated in healthy and dyslexic human subjects in electrophysiological and functional imaging studies using PET, functional MRI (fMRI), transcranial magnetic stimulation (TMS), and magnetoencephalography (MEG) [25, 44–46]. In general, the human MT+ has been assumed to be correlated with the borders of Brodmann areas 19 and 37 or with von Economo and Kostinas’ area OA and PH [47], and is typically found within a dorsal/posterior limb of the ITS, or the junction between this sulcus and lateral/inferior occipital sulcus according to the fMRI results [25, 30, 48, 49]. Human fMRI studies have revealed two distinct subregions, i.e., MT and MST, which are not homogeneous and are arranged in a similar manner as that in the macaquebrain [44]. Receptive field and retinotopic studies showed that MT receptive field constrained mostly to the contralateral visual field [26, 44] and exhibited retinotopic organization [25, 49], whereas MST did not demonstrate retinotopic organization but did respond to peripheral stimuli in both the contralateral and ipsilateral visual hemifields, indicating large receptive fields [25, 44]. The significant characteristics making MST different from MT are the strong responses to ipsilateral stimulation, and have no clear and orderly retinotopic map that MT did contain [25, 50]. The human MST strongly responds to peripheral stimuli with large (contralateral and ipsilateral) receptive fields [25, 50], and also receives vestibular information [51–53]. The physiological properties suggest that human MST is strongly specialized for encoding global flow properties and plays a critical role in the maintenance of smooth pursuit [50, 53, 54].

The arrangement of the two subregions of human MT+ is similar to that in the macaque brain, that is, MT is located at the posterior part of MT+ and MST borders MT area anteriorly [25, 44, 49, 50]. Huk and others located human MST on the anterior/dorsal bank of AOS (also known as the ascending limb of the inferior temporal sulcus), while area MT typically located on the posterior/ventral bank of AOS [25]. The precise position of area MT has been confirmed bycytoarchitectonic study from the Jüelich group [31], but area MST has not been well defined cytoarchitectonically.

Evidence from recent studies on eye movements revealed several features of the pursuit system as functional homologies with saccades [24], and that the overlapping networks between smooth pursuit and saccades include the typical cortical eye fields including the frontal eye field (FEF), supplementary eye field (SEF), dorsolateral prefrontal cortex (DLPFC), parietal eye field (PEF), precuneus and even MT/MST fields [55]. In fact, each of the cortical eye fields is composed of two distinct subregions which are devoted to the control of both saccadic and smooth pursuit eye movements, and has direct projections to neural centers in the brain stem which are involved in eye movement control [23]. Different from the traditional view of pursuit and saccades as distinct oculomotor subsystems, the control of pursuit and saccades might be viewed as different outcomes resulting from a single cascade of sensory-motor functions [24].

Inspired by the physiological and functional evidence from macaque and the human brain, and the precise anatomical localization of the human MST and MT based on the data fromneurophysiologic and functional neuroimaging studies, we hypothesized that the mechanisms of epileptic semiology of ipsiversive eye deviation in the two cases, whose epileptogenic zone has been confirmed to be located in the inferoposterior temporal region, can be explained by involvement of the cortical network of eye movement control, specifically in terms of the smooth pursuit movement.

The two cases we reported here had both similar epileptic semiology and anatomical localization of the epileptogenic zone as ascertained by SEEG. The initial clinical sign of both cases was characterized by forced ipsilateral eye deviation with homodromous head turning, which is similar to the semiology of the case reported by Kaplan [33]. In the present Case1, the epileptogenic zone is located on the posterior ITG and extended to the anterior bank of AOS, which is the precise anatomical location of human MST. In the present Case2, the epileptogenic zone is located on the fusiform and posterior ITG, and the epileptic discharges spread to anterior bank of AOS immediately before the appearance of initial clinical sign. Therefore, the localization of epileptogenic zone in the two cases was of great similarity to the conclusion of Kaplan’s case, whose epileptic seizure originated from right temporo-occiptial cortex [33]. As mentioned above, since area MST strongly responds to visual stimuli in the ipsilateral visual field, epileptic stimulation of MST has the probability to induce ipsilateral conjugate eye deviation, as that manifested by our two cases.

Case1 hadtwo times of SEEG recordingsto determine the exact location of epileptogenic zone and the boundary of cortical resection. Taking all the cortical areas covered in both SEEG recordings into consideration, we had got adequate coverage on cortical eye fields, striate and multiple extra-striate visual cortices. Meta-analysis on all the ictal SEEG of Case1 indicated that the rapid synchronization of high frequency oscillations happened within 400–600 ms among the multiple cortical eyes fields, striate and extra-striate visual cortices including MT+, inferior parietal lobule (IPL), IPS, parieto-occipital sulcus (POS), FEF, and so on. The wide and rapid synchronized ictal epileptic discharges among multiple cortical eye fields are consistent with the viewpoint that the pursuit system has a functional architecture similar to that of the saccadic system [24].

The resemblances of the two cases includeipsiversive eye deviation and the location of epileptogenic zones which were localized in the posterior part of ITG adjacent to AOS—the accurate cortical localization of human MST. According to the characteristics of retinotopic organization in the subregions of MT + and its functional roles in smooth pursuit eye movements, we hypothesize that the lateralization of eye deviation during temporo-occipital epileptic seizures depended on whether MST is involved initially or primarily during the epileptic seizure. Epileptic seizure originated from/primarily involvedthe posterior ITG or anterior bank of AOS (human MST) would probably induce ipsilateral conjugate eye deviation initially.

## Conclusion

To our knowledge, these are the first cases reports focusing on the epileptic ipsiversive eye deviation by using SEEG recordings. The advantages of SEEGinclude itsaccurate cortical mapping and electrode implantation with high spatial resolution on 3D level, and the capacity to sample the cortical activity in the depth of cerebral sulcus. According to the neurophysiologic and functional neuroimaging evidence mentioned above, the core anatomical marker and probable boundary of the cortical location of human MST/MT is the AOS (the ascending limb of the ITS), which had been explored adequately with the exploration of its adjacent and related cortical areas in the two cases. The relationship of exact location of epileptogenic zones of the two cases and AOS convinces us that the manifestation of epileptic ipsiversive eye deviation should be attributed to the neurophysiologic and neuropsychological characteristics of MT+, especially area MST, and its functional role in cortical control of smooth pursuit eye movements.

## Abbreviations

AOS: Anterior occipital sulcus
CT: Computed tomography
DLPFC: Dorsolateral prefrontal cortex
FDG-PET: Positron emission tomography with neurotracer of fluorodeoxyglucose 18 F
FEF: Frontal eyes field
GTCS: Generalized tonic-clonic seizure
IPS: Intraparietal sulcus
ITG: Inferior temporal gyrus
ITS: Inferior temporal sulcus
LOS: Lateral occipital sulcus
MEG: Magnetoencephalography
MRI: Magnetic resonance imaging
MST: Medial superior temporal area
MT: Middle temporal area
MTG: Middle temporal gyrus
PEF: Parietal eye field
POS: Parieto-occipital sulcus
SEEG: Stereoelectroencephalography
SEF: Supplementary eye field
STS: Superior temporal sulcus
TMS: Transcranial magnetic stimulation
