# Erratum to: Ipsiversive ictal eye deviation in inferioposterior temporal lobe epilepsy—two SEEG cases report

**DOI:** 10.1186/s12883-017-0861-y

**Published:** 2017-05-02

**Authors:** Wei Zhang, Xingzhou Liu, Lijun Zuo, Qiang Guo, Qi Chen, Yongjun Wang

**Affiliations:** 1 Department of Neurology, Beijing Tiantan Hospital, Capital Medical University, Beijing, China; 2 Epilepsy Center, Guangdong Sanjiu Brain Hospital, Jinan University, No. 578, Sha Tai Nan Lu, Guangzhou, 510510 China; 3 School of Psychology, South China Normal University, Guangzhou, China; 4 China National Clinical Research Center for Neurological Diseases, Beijing, China; 5 Department of Neurology, Tiantan Clinical Trial and Research Center for Stroke, Beijing Tiantan Hospital, Capital Medical University, Beijing, China; 6 Vascular Neurology, Department of Neurology, Beijing Tiantan Hospital, Capital Medical University, Beijing, China

## Erratum

After publication of the original article [1], it came to the publisher’s attention that a number of corrections submitted by the author during proofing had not been implemented. Upon investigation it transpired that an error in the production process resulted in these further corrections being overlooked.

The overlooked changes were solely to correct typos through the article. For example, in the Background section of the abstract, “thelateralizing” should have read “the lateralizing”. As such, the changes do not affect the scientific content of the article.

The original article has now been updated to rectify the missed corrections requested by the author.
